# Primary care implementation study to scale up early identification and brief intervention and reduce alcohol-related negative outcomes at the community level (PINO): study protocol for a quasi-experimental 3-arm study

**DOI:** 10.1186/s12875-021-01479-9

**Published:** 2021-07-01

**Authors:** Bram Pussig, Lodewijk Pas, Ann Li, Mieke Vermandere, Bert Aertgeerts, Catharina Matheï

**Affiliations:** Academic Centre for General Practice, Kapucijnenvoer 33 blok H - bus 7001, 3001 Leuven, KU Belgium

**Keywords:** Scaling-up, Implementation, Primary healthcare, Hazardous alcohol use, Harmful use of alcohol, Heavy drinking, Training and support, Community action, EIBI

## Abstract

**Background:**

Primary healthcare-based Early Identification and Brief Intervention (EIBI) for hazardous and harmful alcohol use is both a clinically relevant and cost-effective strategy to reduce heavy drinking. Unfortunately, it remains poorly implemented in daily practice. Multiple studies have shown that training and support (T&S) programs can increase the use of EIBI. Nonetheless, gains have only been modest and short-term at best. Suggestions have been made to rely more on multicomponent programs that simultaneously address several barriers to the implementation of EIBI. The PINO-project aims to evaluate the added value of such a multicomponent program to improve EIBI delivery in daily practice.

**Methods/design:**

A quasi-experimental three-arm implementation study in Flanders (Belgium) will assess the effects of tailored T&S to General Practitioners (GPs) with or without community mobilisation on EIBI delivery in general practice. The study lasts 18 months and will take place in three comparable municipalities. In municipality 1 and 2, GPs receive a tailored T&S program. The T&S is theoretically founded and tailored to the GPs’ views, needs and practice characteristics. Furthermore, community actions will be embedded within municipality 1 providing additional, contextual, support. In municipality 3, GPs are offered a minimal intervention to facilitate data collection.

The primary outcome is the proportion of adult patients screened for hazardous and harmful alcohol use at the end of an 18-month implementation period. The secondary outcome is the scaling up activity at municipal level in screening rates, as assessed every 3 months, and the proportion of patients who received an additional brief intervention when necessary. Furthermore, the correlation between the opinions and needs of the GP’s, their practice organisation and their EIBI performance will be explored.

**Discussion:**

The PINO-project addresses the gap between what is theoretically possible and the current practice. This is an innovative study combining T&S at GP level with community actions. At the same time, it implements and evaluates practice T&S based on the theoretical domains framework.

**Trial registration:**

This trial was approved by the Ethics Committee for Research of UZ/KU Leuven (reference number s63342 and G-2020-2177-R2(MAR)) and is registered on clinicaltrials.gov (NCT04398576) in May 2020.

**Supplementary Information:**

The online version contains supplementary material available at 10.1186/s12875-021-01479-9.

## Background

Harmful alcohol consumption is one of the leading risk factors for poor health and death in the world. About 6% of all-cause mortality is attributable to alcohol, which is equivalent to 2.8 million deaths worldwide [1]. In Belgium, 21% of men and 7% women display a hazardous or harmful drinking behaviour [2]. Alcohol-related harm transcends the individual as it also affects families, communities, and health systems [3].

Reducing harmful and hazardous alcohol consumption could have a major impact on lowering the disability-adjusted life years [4]. The majority of alcohol-related health problems occurs in the middle-aged population, suggesting that preventive measures will benefit most when directed at this population [5, 6].

Early Identification and Brief Intervention (EIBI) has been shown to be a cost-effective method to reduce the number of heavy drinking patients in primary care [7]. Interestingly, the highest efficacy occurs when brief advice is delivered to non-treatment-seeking, hazardous drinking patients [8, 9]. Even though EIBI in primary healthcare was found to be effective, and general practitioners (GPs) recognize that primary healthcare is an important and appropriate setting for delivering EIBI, implementation of EIBI is generally lacking [10]. Considerable effort has been invested in identifying barriers to explain this observation [11, 12]. Lack of resources, workload, and absence of Training and Support (T&S) are identified as the main implementation barriers, whereas adequate resources and training are considered the main facilitators [13]. The evaluation of different strategies addressing these barriers with a view on improving implementation of EIBI by GPs yielded only modest results [14].

Previous strategies that aimed to upscale EIBI delivery in general practice remediated only a limited number of barriers and relied on a one-size-fits-all approach [15]. Such a basic approach is likely to be ineffective in the highly variable and complex context of general practice, which is marked by differences in practice organisation, personal needs, believes and attitudes [16]. Multicomponent programs, adapted to the needs and concerns of both GPs and the general population, regarding alcohol-related EIBI implementation is advised [17–19]. Public health interventions directed at the general population, the so-called ‘community actions (CAs)’, are considered essential to facilitate EIBI delivery in general practice [20]. CAs have been suggested to reframe the public’s and professionals’ views on alcohol use [20]. Unfortunately, to date, integrating such CAs to facilitate EIBI delivery has only generated limited attention [20].

T&S have been found to increase EIBI rates for GPs who feel confident and are committed to work with at-risk drinkers. Training programs can significantly improve providers’ knowledge, self-efficacy, and expectations about the value of EIBI [21–23]. However, it does not affect less secure or motivated GPs to the same extend [18]. These findings underline the need to address GPs’ attitudes, needs and views towards working with at-risk drinkers in training and support programs. Customising support with personal experiences should motivate GPs and create opportunities to scale up EIBI-performance [24, 25]. Furthermore, compatibility with the actual practice activity should be considered [26], including timing and the choice of innovation steps [24].

Poor sustainable results in implementing EIBI might also be due to the lack of a sound theoretical implementation model. Currently, the most comprehensive model is the Behavioural Change Wheel (BCW) linked to the Theoretical Domains Framework (TDF). The TDF provides a comprehensive assessment of the factors that are likely to influence practitioners’ target behaviour [27]. Furthermore, the TDF can be used as a theoretical model to address the barriers and facilitators for EIBI delivery in general practice by its mapping of related behavioural changes techniques [12].

## Aim and objectives

The PINO-project, which stands for Professional Involvement to reduce alcohol Negative Outcomes, aims to scale up the use of EIBI for hazardous and harmful alcohol use in general practice. This study is based on the hypothesis that training and supporting GPs, tailored to their beliefs, attitudes, and working context, together with community mobilisation may improve their EIBI performance. The PINO-project has the following objectives:

1. To implement and evaluate a multicomponent program comprising tailored T&S and CAs to increase the delivery of EIBI in general practice over an 18-month period.
2. To study the added value of integrating CAs within the strategy to improve EIBI delivery in general practice.
3. To monitor individual GPs’ progression on EIBI delivery for hazardous and harmful alcohol use on a 3-monthly basis and evaluating the effect of scaling up activities on municipal level (e.g., tailored support, CAs).
4. To assess EIBI performance at practice level in relation to the characteristics of GPs and their practice organisation, the changes in GPs’ beliefs and attitudes and the provided support.

## Methods

### Study design and setting

The PINO-study consists of a quasi-experimental three-arm implementation study in three comparable medium-sized Flemish (Belgium) municipalities [28]. All three municipalities have a multicultural population with around 80,000 to 100,000 inhabitants. In addition, they are all marked by a large student population due to the presence of universities or university colleges [28].

#### Municipality 1

Intervention area where GPs receive T&S embedded within a community mobilisation program.

#### Municipality 2

Intervention area where GPs receive T&S without CA.

#### Municipality 3

Support As Usual (SAU) area where GPs receive a minimal intervention that comprises an overview of the current guidelines and knowledge.

**Fig. 1:**
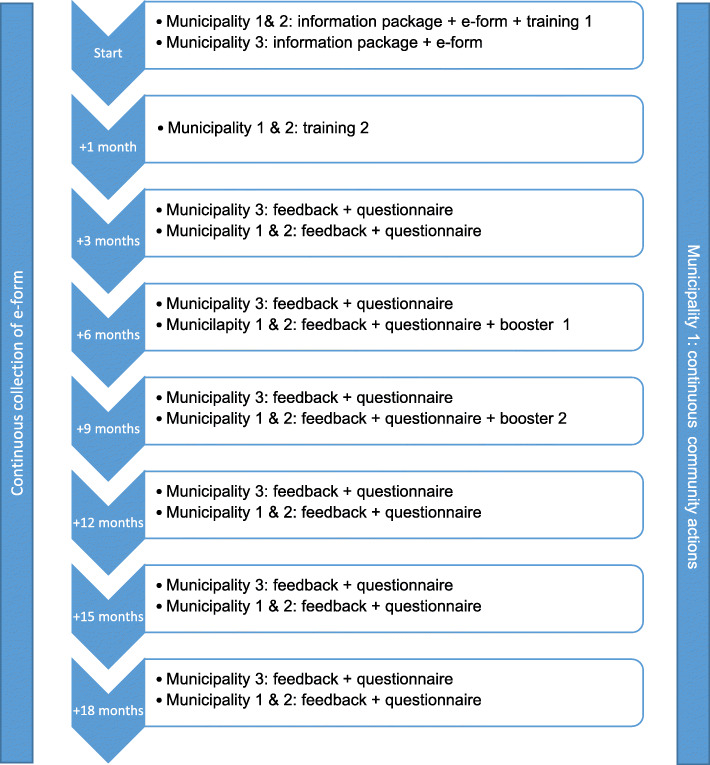
Timeline of the study

During an 18-month implementation period, GPs in the T&S area’s will receive personalised support linked to an online evaluation of their needs, views, practice organisation and EIBI-performance. This on a three-monthly base (Fig. 1).

### Outcomes

The primary outcome comprises the overall early identification-rates of GPs during the 18-month period, specified per municipality. This is the proportion of the consulted adult patients (> 18 years) attending general practice for any reason, which were questioned about their alcohol consumption over an 18-months period at area level.

The early identification rates will be measured as follows:

- **Numerator:** Patients (> 18 years) attending general practice for any reason who are questioned about their alcohol consumption during the study period of 18 months per area.
- **Denominator:** Number of unique patients (> 18 years) attending general practice during the 18 months study period per area.

Secondary outcomes will be related to personal views, as measured using the TDF domains, to practice organisation and to provided support. These secondary outcomes comprise (a) the cumulative early identification rates and (b) the cumulative brief intervention rates.

#### Cumulative early identification rate

The proportion of patients that visited the general practitioner’s office for any reason that were questioned about their alcohol consumption at 3, 6, 9, 12, 15 and 18 months after baseline at area level:

- **Numerator:** Patients (> 18 years) attending general practice for any reason that were questioned about their alcohol consumption during the periods 0–3, 0–6, 0–9, 0–12, 0–15 and 0–18 months per area.
- **Denominator:** Number of unique patients (> 18 years) attending general practice during the periods 0–3, 0–6, 0–9, 0–12, 0–15 and 0–18 months per area.

#### Cumulative brief intervention rate

The proportion of patients (> 18 years) screening positive for hazardous or harmful alcohol use that received oral brief advice/intervention and/or were referred to a digital-based system for advice and/or were referred to another healthcare provider during the periods 0–3, 0–6, 0–9, 0–12, 0–15 and 0–18 months after baseline at area level.

- **Numerator:** Number of unique patients (> 18 years) that received oral brief advice/intervention and/or were referred to a digital-based system for advice and/or were referred to another healthcare provider during the periods 0–3, 0–6, 0–9, 0–12, 0–15 and 0–18 months per area.
- **Denominator:** Number of unique patients (> 18 years) screening positive for hazardous or harmful alcohol use as assessed for 0–3, 0–6, 0–9, 0–12, 0–15 and 0–18 months per area.

### Recruitment and eligibility criteria

All GPs within the three municipalities will be addressed systematically through the local GP associations by means of a standard information letter. In addition, a researcher will personally contact the GPs by telephone for clarifying the project and providing reminders. Both GPs and GP trainees will be invited to participate. GPs will be eligible for inclusion if they can read and understand Dutch, and if they work in a general practice within one of the three included municipalities.

GPs sign an ICF specifying their agreement to participate in all trainings, answer the needed online questionnaires and record screening and advise activities in e-forms. E-forms are electronic forms, integrated in the Electronic Health Records (EHR) of GPs. They are developed to record EIBI delivery, diagnosis and referral. There will be no remuneration provided for the work as it is consistent with regular quality practice. All included practices will need to display a poster in the waiting room, indicating their participation in the study. The poster explains that the practice is taking part in a study that evaluates anonymized data from patient records for scientific purposes. Patients may oppose to being included in the study upon simple request.

### Interventions

#### Minimal intervention

All GPs in the comparator group (municipality 3) will receive an information package containing the most current recommendations by the Flemish GP association concerning harmful and hazardous alcohol use [29]. These recommendations include the guidelines of the Superior Health Council of Belgium^1^ regarding responsible alcohol use, i.e., an acceptable weekly intake of maximum 10 units of alcohol per week with a least two alcohol-free days [30]. The package also comprises educationally conceived practice tools for EIBI, referral strategies to specialised services or internet support about EIBI and links to online documentation.

#### Training and support

The T&S provided in this study will be facilitated by a team of researchers with both international and local experience related to alcohol use in general practice. The trainers are affiliated with the faculty of medicine from the KU Leuven and have an active role in vocational training and continuing medical education for GPs and GPs in training.

##### Training

At the start of the study, GPs in municipality 1 and 2 will receive a standardised educational training based on the TDF-concepts to increase capability, motivation and opportunities for the delivery of EIBI. Here, the differences in practice organisation will be emphasized. Two booster sessions will be planned at 6 and 9 months after baseline (Table 1). These booster sessions will provide support based on changes in the TDF domains, grouping GP in relevant homogenous groups. Due to the SARS-CoV-2 pandemic, a fully online blended learning training package was designed comprising two preparatory online e-learning modules followed by two livestream sessions. If possible, the booster sessions will be performed face to face.

**Table 1:**
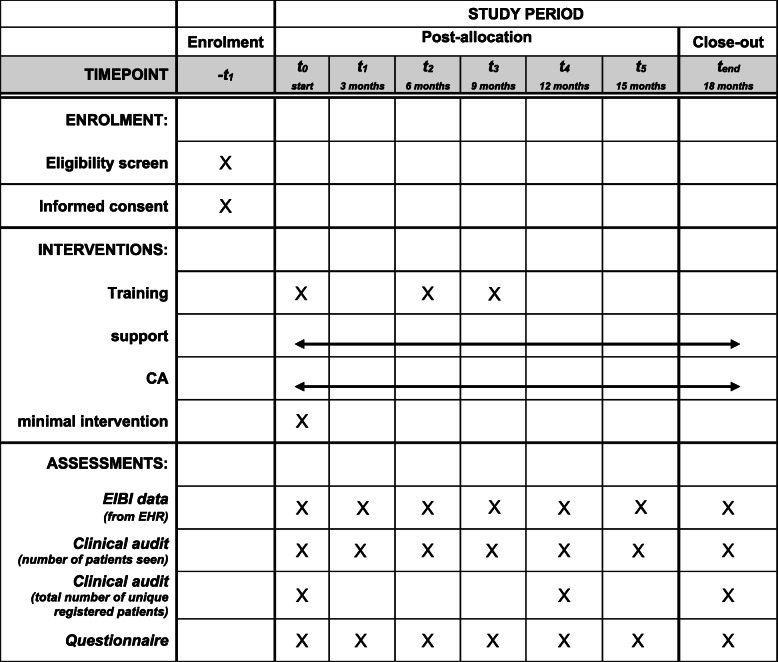
SPIRIT flowchart

*CA* community actions, *EIBI* Early Identification and brief interventions, *EHR* electronic health record

Personalisation of the training package will be established by assessing changes in capability, motivation, believes and practice organisations of the participating GPs. Therefore, standardized online inquiries [31] and active interaction during the livestream sessions will be used. The content of each training is based on previously conducted studies on the subject matter [32, 33] and have been piloted in training practices outside the intervention areas. The training sessions will aim to encourage experience-based reflection related to EIBI delivery, GP’s attitudes and highlights practice organisation. During these sessions, skills will be practiced and reflections on how to implement EIBI in relation to practice characteristics will be stimulated. Further, task definition and delegation of possible tasks, management of EHR and other features of practice organization and collaborative care will be emphasized during these training sessions. Importantly, this training package combines theory and practical exercises facilitated by acknowledged and experienced trainers.

##### E-learning modules

Two e-learning modules were developed on the SOFIA learning platform (ACCO, Leuven, Belgium). This platform includes various didactic functionalities (text, audio, video) to convey information. Furthermore, it allows the integration of inquiries or free text fields for participants to reflect on personal experiences, needs or concerns.

The first e-learning module addresses the risks related to alcohol use, and the current guidelines. In addition, tools for EIBI on alcohol consumption will be presented. The focus here is on how to start a conversation on alcohol use, how to use the Alcohol Use Disorder Identification Test(−Consumption) (AUDIT(−C)), and how provide simple or brief advise to patients according to the possibilities. This first e-learning module was estimated to take 50 min to complete.

The second e-learning will elaborate on how to provide a brief intervention, how to facilitate referral and how to deal with heavy alcohol use or dependence. The importance of a well-defined action plan regarding practice management will be discussed. This e-learning module was estimated to take 30 min to complete.

##### Livestream sessions

Two online interactive training sessions, 90 min each, will be organised via ZOOM (Zoom Video Communications Inc., CA, USA). Both training sessions will proceed on the concordant e-learning modules and are structured by means of a slideshow. During these sessions, emphasis lies on practical exercises and on sharing experiences, problems and solutions. In order to facilitate these sessions, breakout rooms will be used to divide the GPs into smaller virtual groups where they can practise the application of EIBI through role playing and discuss it among themselves. GPs will be grouped based on their practice organisation to allow group discussions in which all GPs can participate equally while also creating a more personalised setting. Attention will be given to possible choices in the task definition, practice organization, and collaborative care, in accordance with the TDF and BCW.

The booster training sessions will start with GPs sharing their personal experiences, perceived barriers and facilitators concerning EIBI delivery. Participants will be grouped according to their similarities in TDF domains and their EIBI activity. This will be measured by preceding online inquiries and by evaluating the use of the e-forms respectively. The content of these sessions will be adapted based on the responses of the TDF inquiries [12, 34]. GPs will be stimulated to discuss problems met with experienced trainers and to share good practices with the group.

### Support

The GPs will receive ongoing support through individual feedback concerning their EIBI-performance, their expressed needs and views, and practice organisation. This on 3-monthly intervals (Table 1). This well-established, evidence-based strategy for quality assurance [19] will be implemented only in the two T&S areas (municipality 1 and 2).

#### Community actions

In their Phase IV study, the WHO suggests that an additional beneficial impact of CA on EIBI uptake is possible, although conclusive empirical data is not yet available [20]. Therefore, we will assess the effect of tailored T&S in a municipality with CA to reframe public and professional views on alcohol (municipality 1) and compare it to municipalities without CA (municipality 2 and 3). These actions will include, but will not be limited to, mass media information campaigns, awareness-raising activities and local neighbourhood mediated actions.

##### Neighbourhood-mediated actions

Neighbourhood-mediated actions will be organised to address the community’s need to initiate the discussion on alcohol outside the clinical setting [35]. The content of these actions will be customised to the neighbourhood’s needs and concerns and presented by local community champions. These champions are locally acknowledged key people in the communities capable of enabling a cultural shift in favour of the wanted behaviour by promoting important values of the intervention [36, 37]. The research team will provide the needed scientific support and means to organise the actions. Examples of such actions will include debates at community centres, mocktail workshops with informative panels, information sessions or a drink-pouring exercise [38].

##### Mass media

The aim of this mass media campaign is trifold [35]. First, it will address individuals’ behaviour towards alcohol use by increasing knowledge on alcohol-related harms and by creating awareness one’s alcohol consumption. Second, this campaign will motivate the community to discuss alcohol use with their GP. Third, it will support neighbourhood-mediated actions. Various means of mass communication including printed-, broadcast- and social media will be used to convey these messages.

### Timeline

The intervention phase commenced in January 2021 with the recruitment of GPs. Recruitment is spread over 4 months with 1 month difference in the three areas. The study will run for 18 months after baseline assessment (Fig. 1).

### Statistical methods

#### Sample size estimation

The primary research question, driving the sample size calculation, is whether the provision of tailored T&S to GPs alone or in combination with CAs will increase the early identification of hazardous and harmful alcohol use among consulting adults in general practices.

The study is powered to detect a statistically significant difference in the cumulative screening rate over the 18-month intervention period. Based on the results of the ODHIN study, we expect the intervention (tailored training and support to GPs) to achieve a rate of screening that is at least 12% or a doubling of the actual screening rate that is assumed [39]. For an alpha of 0.05 and 80% power, a total sample size of 1167 adult patients (389 in each group) is estimated.

To account for a clustering effect at the primary care practice level, with an intra-cluster correlation coefficient (ICC) set at 0.03, based on the findings of the ODHIN study, and a mean cluster size of 2256 patients (2.35 GPs per practice each seeing 960 adult patients per 3 months), the number of patients required was multiplied by 68.65 corresponding to the cluster design effect (DE = 1 + ICC (size of the cluster-1)). Thus, the final sample will consist of 80.115 patients (26.705 per arm), and 84 GPs are needed (28 in each region).

Although conclusive empirical data on the impact of CA is not available, a combined intervention-effect of the mean practice screening rate was set at 20%. This was based on practical experiences and international discussions with other researchers in the field and relates to the needed effect size to consider CA worthwhile. To detect a difference in screening rate of 8% between municipality 1 and municipality 2, we would have a power of 87%.

### Statistical analysis plan

Baseline characteristics of the participating GP population will be compared for the 3 arms including practice (practice size, multidisciplinary practice, practice management EIBI) and GP characteristics (gender, training background, and TDF domains).

The primary outcome of the study will be the proportion of consulting adult patients screened, measured over the 18-month intervention period. The primary endpoint will be analysed according to the intention-to-treat approach. Therefore, linear regression will be used for our data analysis with early identification rates over the 18-month intervention period as a continuous outcome and the intervention group as a factor. Adding a random effect in the model will deal with clustering by general practice. The group effect will be reported as a relative risk with a 95% confidence interval. Secondary outcomes will be analysed similarly.

Subgroup analysis will allow to investigate how the primary outcome behaves in function of covariates. Here, the hierarchical nature of the data and characteristics at different hierarchy levels (i.e. characteristics of the GPs, characteristics of the practices) will be taken into consideration. Participants will be compared for primary and secondary outcomes according to changes in views as measured by TDF domains and practice organisation, taking into account the presence of CAs.

A distinction between active GP participants from less active GP participants will be made by listing those who used the AUDIT(−C) e-forms at least once versus those who did not use these forms.

#### Handling of missing data

If a GP withdraws from the study prematurely, all data collected until that point will be analysed (using the intent-to-treat approach). No additional data will be collected after withdrawing from the study.

#### Data collection

##### EIBI data

After signing an Informed Consent Form (ICF), all GPs in the three municipalities will have access to an e-form embedded in their EHR. This is a clinical data collection form accessible through the EHR, permitting the standardized introduction of screening results from the AUDIT(−C), alcohol-related diagnoses and actions (e.g., the provision of verbal, brief advice/intervention, referral to a digital system for advice or referral to another healthcare provider). At each contact, when they fill in their e-forms, GPs should validate the form and transmit the content in an encrypted format.

Collection of these anonymized patient data will occur via a double secured server to enable safe data transfer and provide a secure environment for data handling and data analysis for research purposes under Belgian legislation [40]. The data protection officer of the KU Leuven will monitor this process.

##### Clinical audit data

For every participating GP, the following data will be collected through a clinical audit of their EHR (Table 1):

- The number of patients seen per practice and GP over the past 3 months at 0, 3, 6, 9, 12, 15 and 18 months;
- The total number of unique patients registered with a GP or practice at 0, 12 and 18 months

GPs send clinical audit data to a double secured server at the Academic Centre for General Practice (ACHG), as authorized in the ICF. The data remain linked to the National Health Services Coding of the consulted GP (NHS-Code).

##### Online GP enquiry

Four times a year, GPs will receive an online questionnaire to (Table 1):

- Identify positive and negative experiences
- Assess role security and therapeutic commitment of the participating GPs using the Short version of the Alcohol Attitudes Problems Perception Questionnaire (SAAPPQ) [41].
- Measure views based on TDF domains in relation to knowledge and skills for EIBI, personal priority setting, peer’s views, motivation, readiness for change, and emotional coping.
- Identify T&S needs.
- Obtain insights into created opportunities and changes in practice approach (e.g., case findings versus screening, targeted or universal, application of task delegation, waiting room materials, or facilitating internet access).

Every three months, an integrated dataset, linked with NHS-codes, will be created containing:

1. Absolute number of EIBI per age group, gender, area, and GP
2. Clinical audit data of the adult population stratified for gender and age-groups per area and GP
3. GPs characteristics, views, experiences, practice management and needs per area and GP
4. T&S received per GP in the intervention areas

The data will be presented as feedback to the GPs for benchmarking by area in municipality 1 and 2. In order to compare primary and secondary outcome measures, at patient counts per area level, EIBI rates will be grouped per area. Furthermore, a comparison will be made between age groups, gender, and co-morbidity since the start of the study.

## Discussion

The strength of this study is the combination of training and interactive support, tailored to a theory-based implementation strategy (TDF and BCW) and measured during the implementation process. PINO addresses both in- and external barriers and facilitators. This will increase GPs’ experience according to their daily practice and develop good experiences by learning from less successful ones to obtain sustained behavioural and practice change. What also distinguishes PINO from other studies is the inclusion of CAs and evaluating their added value on the delivery of EIBI in general practice.

The PINO-project has some potential limitations. There is a possibility for selection bias within the recruitment of GPs. Highly motivated GPs are more likely to participate compared to more sceptical or less motivated GPs. Nonetheless, a quasi-experimental design is the right fit for this project due to its pragmatic character and the strong external validity [42]. In order to minimize potential bias, necessary precaution was taken when designing the study (e.g., including a control area, using appropriate analytical techniques). Additionally, GPs may address the subject of alcohol consumption but forget to register it in their EHR. It is also possible that GPs remember to register, but do not fill in the e-form correctly. These problems should however occur less in the cities where GPs receive T&S. These concerns have been taken into account while designing this study by integrating both e-forms as well as clinical auditing of the EHR. The e-form provides GPs the opportunity to discuss and systematically record how much alcohol their patients use. Another matter to consider is the possibility that data collection is incomplete due to technical errors, which will be take into account during the analysis.

Nevertheless, the strengths of this study outweigh its limitations. PINO is based on theoretical models of implementation and includes theory-based interventions to promote the desired behaviour. It addresses the attitudes of GPs towards working with at-risk drinkers, and it tailors its program to their views and needs.

## Supplementary Information

**Additional file 1.** Informed consent file PINO-project (Dutch).

## Data Availability

The research team will have access to interim results and the final data set. Every three months, the AB Inbev Foundation will receive aggregated interim results. The study results will be disseminated via open-access, peer-reviewed publications and conference presentations. Study materials are available on request. This protocol is written according to the SPIRIT guidelines for clinical trial protocols [43].

## Abbreviations

ACHG: Academic Centre for General Practice
AUDIT: Alcohol Use Disorder Identification Test
AUDIT-C: Alcohol Use Disorder Identification Test - Consumption
BCW: Behavioural Change Wheel
BI: Brief Intervention
CA: Community Action
DPO: Data Protection Officer
EC: Ethics Committee
EHR: Electronic health record
EIBI: Early Identification and Brief Intervention
GP: General Practitioner
NHS: National Health Service
SARS-CoV 2: Severe Acute Respiratory Syndrome Corona Virus 2
T&S: Training and Support
TDF: Theoretical Domains Framework

## Footnotes

1 The Superior Health Council of Belgium is the highest healthcare policy-authority responsible for giving scientific advice to the Belgian federal government.
