# Supplementary material for: Primary care implementation study to scale up early identification and brief intervention and reduce alcohol-related negative outcomes at the community level (PINO): study protocol for a quasi-experimental 3-arm study
Source: BMC Fam Pract. 2021 Jul 1;22:144. doi: 10.1186/s12875-021-01479-9 (PMC8248288; doi:10.1186/s12875-021-01479-9)
Supplement: Supplementary file 1 — Additional file 1. Informed consent file PINO-project (Dutch). [file 12875_2021_1479_MOESM1_ESM.pdf]

Inlichtingen- en toestemmingsformulier voor deelname aan onderzoek:

Implementatie studie om alcohol gerelateerde vroeg-detectie en kort advies te stimuleren in de eerstelijnszorg met als doel overmatig alcoholgebruik te verlagen in de gemeenschap: Het PINO-project

**Onderzoekers:**	**Leo Pas, Bram Pussig,**
**Hoofdonderzoekers:**	**Cathy Matheï, Mieke Vermandere, Bert Aertgeerts**
**Onderzoekscentrum:**	**Academisch Centrum voor Huisartsgeneeskunde**
	**Kapucijnenvoer 33, blok J, PB 7001 - 3000 Leuven**
**Opdrachtgever:**	**KU Leuven**
**Financiering:**	**AB Inbev Foundation**
**Ethische commissie:**	**Ethische Commissie Onderzoek UZ/KU Leuven**

Overzicht van wijzigingen aan het document

Versie nummer	Publicatiedatum	Beschrijving aanpassing
Versie 1	05/11/2019	/
Versie 2	20/12/2019	Aanpassingen gerelateerd aan de anonimiteit van de patiëntgegevens
Versie 3	30/01/2020	Toevoegingen in functie van AVG-wetgeving<br>Verduidelijking over rol van Huisarts als deelnemer<br>Toevoeging van DPO
Versie 4	10/03/2021	Aanpassing van de deelnemende regio's.

Met wie kan ik contact opnemen als ik vragen heb?

Naam	Functie	Voor	Contact-gegevens
Cathy Matheï	Hoofdonderzoeker van het studiecentrum	Informatie, problemen, bezorgdheden	+32 16 37 72 96<br>**E-mail:** catharina.mathei@kuleuven.be
	Studiepersoneel	Informatie, problemen, bezorgdheden	+32 16 32 51 42
	Data protection officer (DPO)	Problemen, onduidelijkheden over de verzamelde gegevens.	dpo@kuleuven.be
	Contact voor dringende gevallen	Noodgeval	+32 16 32 51 42
	Ombudspersoon patiëntenrechten	Bezorgdheden over je rechten als deelnemer aan een studie	privacy@kuleuven.be
	Belgische gegevensbeschermings autoriteit	Klachten over de vertrouwelijkheid van je gegevens	**E-mail:** contact@apd-gba.be

Beschrijving van de studie en je rechten bij deelname

1. Waarom doen we deze studie?

Geachte dokter

Eén kwart van de Vlaamse mannen en 10% van de Vlaamse vrouwen zou naar schatting regelmatig te veel alcohol drinken. Vroegdetectie en kort advies, verzorgd via de eerstelijnszorg, blijken effectief te zijn in de aanpak van dit overmatig alcoholgebruik. Echter is gebleken dat slechts één op vijf van de overmatig alcohol drinkers een gepast advies of behandeling krijgt. Er werd reeds veel onderzoek gedaan om te achterhalen wat de barrières en faciliterende factoren zijn die vroegdetectie en kort advies beïnvloeden. Op basis van deze factoren werd binnen het Academisch Centrum voor Huisartsgeneeskunde (ACHG) het PINO-project opgestart. Dit project tracht te achterhalen hoe vroeg-detectie en kort advies optimaal bevorderd kan worden.

Het PINO-project staat voor 'Professionele Inschakeling om de Negatieve effecten van alcoholgebruik Om te buigen'. Het is een praktijk bevorderend onderzoek met als **Doel** vroegdetectie en kort advies, in verband met overmatig alcoholgebruik, te verbeteren in de huisartspraktijk.

Voordat je beslist over jouw deelname aan deze studie willen we je wat meer informatie geven over wat dit juist betekent op organisatorisch vlak en wat de voor- en nadelen zijn van jouw deelname. Zo kan je een beslissing nemen op basis van juiste en volledige informatie. Wij vragen dat je de volgende pagina's met informatie aandachtig te lezen. Je kan met al jouw verdere vragen terecht bij de onderzoekers waarvan de contactgegevens bovenaan dit document terug te vinden zijn. Op het einde van dit formulier kan je dan jouw toestemming voor deelname bevestigen.

2. Waarom wordt mij gevraagd deel te nemen?

Dit project is een praktijk-bevorderend implementatieonderzoek. Je wordt gevraagd om deel te nemen aan dit onderzoek omdat we op zoek zijn naar geïnteresseerde huisartsen die samen met ons willen werken om de praktijkvoering in de huisartsgeneeskunde te bevorderen.

Voor dit project werden enkele criteria opgesteld waaraan voldaan moet worden om aan het project te kunnen deelnemen:

- ✓ Je bent huisarts of huisarts in opleiding (HAIO)
    - Je kan deelnemen met meerdere of al de collega's uit jouw praktijk of als individuele arts. Praktijkopleiders en hun HAIO's dienen samen in te schrijven.
- ✓ Je praktijk of groepspraktijk is gevestigd in:
    - Leuven, Mechelen of Hasselt (of een van de deelgemeenten)
- ✓ Je moet de Nederlandse taal machtig zijn (spreken, schrijven en lezen)

Daarnaast werden er ook criteria vastgelegd die het niet mogelijk maken om deel te nemen aan de studie.

- Wanneer het niet mogelijk is om het E-form te integreren in het Elektronisch Medisch Dossier (EMD). Dit zal gecontroleerd worden op basis van de eerste, voorafgaande, online bevraging voor de start van de studie.

3. Moet ik deelnemen aan het onderzoek?

Je deelname aan een studie gebeurt vrijwillig en mag nooit onder druk gebeuren. Dit betekent dat je het recht hebt om niet deel te nemen aan de studie. Je mag je ook op elk moment terugtrekken zonder dat je hiervoor een reden hoeft te geven, zelfs al heb je eerder toegestemd om deel te nemen. We vragen om de onderzoekers op de hoogte te brengen van je beslissing. Je beslissing zal geen invloed hebben op je relatie met de onderzoekers, noch op de kwaliteit van mogelijke toekomstige samenwerking.

4. Wat zal er tijdens de studie gebeuren?

Verloop: Dit is een quasi-experimentele studie die in 3 vergelijkbare gemeenten (Leuven, Mechelen en Hasselt) gedurende 24 maanden loopt (3 maanden opstart, 18 maanden opvolging, 3 maanden nameting). In de deze gemeenten worden verschillende ondersteunende methoden onderzocht met als doel ze te vergelijken op hun efficiëntie om alcohol gerelateerde vroegdetectie en kort advies te bevorderen in de huisartspraktijk. Door een verschil in te bouwen in de ondersteuning van huisartsen kunnen we nagaan wat het beste helpt om vroegdetectie en kort advies, in verband met alcoholgebruik, te bevorderen. In Leuven worden praktijkondersteuning en trainingen samen met gemeenschapsondersteuning aangeboden, in Mechelen is er praktijkondersteuning en training voorzien voor de huisartsen en in Hasselt wordt de standaard ondersteuning gehanteerd.

Training en ondersteuning

Voor de start van de 18 maanden opvolgperiode zullen twee educatieve trainingsmomenten gepland worden. Deze trainingen zullen herhaald worden na 6 en 12 maanden, gepersonaliseerd aan jouw ervaringen, attitudes en noden. Deze noden worden bepaald aan de hand van een online bevraging. De online bevraging zal gedurende het onderzoek in meer beperkte vorm herhaald worden om gepersonaliseerde opvolging te garanderen.

Tijdens deze trainingen zullen theoretische kennis, vaardigheden, attitudes, ervaren barrières en faciliterende factoren, en de vooruitgang van je persoonlijke aanpak om vroegdetectie en kortadvies te implementeren besproken worden. Een training duurt ongeveer 2 uur.

Driemaandelijkse feedbackmomenten worden voorzien over de bereikte en geadviseerde doelgroep.

Gemeenschapsondersteuning

Om de bespreekbaarheid van alcohol te verhogen in de huisartspraktijk zullen enkele gemeenschap-georiënteerde acties gepland worden in Leuven en haar deelgemeenten. Deze acties zullen in de mate van het mogelijke aangepast worden naar de noden van de gemeenschap. Je zal als huisarts op de hoogte gebracht worden van de specifieke acties die ondernomen worden.

Standaard ondersteuning

Voor de standaard ondersteuning worden de richtlijnen en bruikbare tools, beschikbaar bij relevante instanties zoals het VAD en DomusMedica, aangereikt.

5. Zal ik voordeel halen uit de studie?

De informatie die tijdens de studie verkregen wordt zal bijdragen tot een verbeteren van de inzichten om de opsporing en bespreking van risico gebruik van alcohol in de huisartsgeneeskunde. Je deelname zorgt voor een bijdrage aan het bevorderen van de huisartsgeneeskunde en je persoonlijke praktijkorganisatie.

6. Welke zijn de mogelijke risico's bij deelname aan de studie?

Er zijn geen risico's verbonden bij deelname aan deze studie. Het kan zijn dat er tijdens de bevragingen persoonlijke of gevoelige informatie bevraagd wordt. Je bent niet verplicht om op vragen te antwoorden waar je je oncomfortabel bij voelt of die te persoonlijk zijn. Het niet antwoorden op vragen heeft geen gevolgen voor verdere contacten of deelname aan studies.

7. Kan mijn deelname aan de studie vroegtijdig eindigen?

Zoals verder in dit deel gedetailleerd besproken wordt, kan je deelname aan de studie vroegtijdig eindigen wanneer

- je besluit je toestemming in te trekken,
- de onderzoekers besluiten je deelname aan de studie te stoppen.

De opdrachtgever kan gegevens die reeds werden verzameld vóór de beëindiging van je deelname, blijven bewaren en gebruiken. Dit is bedoeld om een foutieve interpretatie van de studieresultaten te vermijden.

Je besluit je toestemming in te trekken

Je hebt het recht om je toestemming zonder een reden in te trekken. Wel vragen we je de onderzoekers op de hoogte te brengen van je beslissing. Ook al is het niet verplicht, kan het voor de onderzoekers en opdrachtgever nuttig zijn de reden voor je beslissing te kennen. Wanneer je beslist om uit het onderzoek te stappen, zullen alleszins geen nieuwe gegevens over je praktijkvoering meer verzameld worden.

De onderzoekers besluiten je deelname aan de studie te stoppen

Jouw deelname aan deze studie kan ook worden beëindigd door de onderzoekers omwille van belangenvermenging of als gemaakte afspraken niet worden gerespecteerd. De reden hiervoor zal altijd uitgelegd en besproken worden met de onderzoekers.

8. Zal mijn deelname aan de studie bijkomende kosten met zich meebrengen voor mij?

Jouw deelname zal geen bijkomende kosten met zich meebrengen. Deelname aan deze studie is op vrijwillige basis. Verplaatsingsvergoedingen zullen voorzien worden.

9. Welke gegevens worden over mij verzameld tijdens de studie en wat gaat ermee gebeuren?

Welke gegevens worden tijdens de studie verzameld en verwerkt?

In het kader van het onderzoek zullen enkele gegevens verzameld worden via verschillende methoden. Deze gegevens zijn nodig om een antwoord te genereren op wat er onderzocht wordt. Deze gegevens omvatten geanonimiseerde patiëntengegevens (zoals hieronder beschreven) die gelinkt worden aan jouw persoonlijk RIZIV-nummer. Dit is nodig om de nodige opvolging en feedback te kunnen genereren tijdens de studie. De onderzoekers hebben geen toegang tot identificerende gegevens van je patiënten en kunnen enkel de link maken tussen u (als arts) en de anonieme gegevens uit het EMD en e-form zoals hieronder beschreven. Al de gegevens worden behandeld volgens de AVG-wetgeving.

De gegevens zullen verzameld worden door middel van een klinische audit, E-form en online bevragingen.

- ➢ Klinische audit

De volgende data wordt verzameld doormiddel van een klinische audit van het Elektronisch Medisch Dossier (EMD). We zullen je tevens helpen voor uitvoeren van een klinische audit van jouw praktijkpopulatie bij start en met driemaandelijkse intervallen. In de opleiding worden hiervoor de nodige vaardigheden aangeleerd en ondersteuning is nadien verder beschikbaar. Je krijgt hierdoor meer inzicht in hoe jouw praktijkaanpak werkt of kan verbeteren.

  - Het aantal patiënten gezien in de praktijk.
  - Het totaal aantal uniek geregistreerde patiënten bij u of uw praktijk.
  - Dossier vermelding van alcoholgebruik.

- E-form

Voor elke gescreende patiënt voor overmatig alcoholgebruik zal een E-form vervolledigd moeten worden waar onderstaande data van de patiënt in beschreven moet worden. Een E-form is een in jouw dossiersysteem toegevoegd formulier om de gegevens nodig voor een goede zorg uniform te registreren. De huidige dossiersystemen laten immers onvoldoende de standaardisatie van de gegevens over alcoholgebruik toe. Met een "*" gemerkte gegevens worden automatische uit jouw dossier opgenomen bij het openen van dit formulier

  - Geboortejaar patiënt*
  - Geslacht patiënt*
  - RIZIV-code arts*
  - Contactdatum*
  - Relevante medische diagnoses *
    - Actieve en passieve ICPC-codes*

Het formulier helpt je om een gestandaardiseerde inschatting te maken van volgende gegevens:

  - AUDIT-C-score
  - AUDIT-score (indien AUDIT-C groter of gelijk is aan 5)
  - Jouw beoordeling van het alcoholgebruik patroon van de patiënt
  - Jouw advies of verwijzing

- Online bevraging

Een online bevraging wordt in meerdere keren, in beperkte vorm, herhaald om de verandering in noden en praktijkaanpak op te volgen. Deze gegevens zijn essentieel voor de personalisatie van de trainingen en ondersteuning.

Hoe zullen de onderzoekers de gegevens behandelen?

De onderzoekers zijn gebonden door het beroepsgeheim bij het verzamelen en verwerken van je gegevens. Dit betekent dat hij/zij je identiteit nooit zal bekendmaken, ook niet in een wetenschappelijke publicatie of een voordracht, en dat hij/zij je gegevens zal coderen (d.w.z. je identiteit in de studie vervangt door een identificatiecode) alvorens ze naar de opdrachtgever te sturen. Daardoor zullen de onderzoekers, en het studiepersoneel onder de verantwoordelijkheid van de onderzoekers, de enigen zijn die je identiteit zullen kunnen koppelen aan de gegevens die tijdens de studie zijn doorgegeven.

De patiëntgegevens die verzameld worden, zullen geanonimiseerd aan de onderzoekers bezorgd worden. Dit zal gebeuren via een versleutelde overdracht via een door de overheid erkende partner (e-health). De geanonimiseerde data zijn enkel beschikbaar voor de betrokken onderzoekers. Jouw

praktijkgegevens worden gekoppeld aan jouw RIZIV-nummer en je identiteit zal op geen enkel moment vrijgegeven worden buiten deze onderzoeksgroep. Eventuele referenties, in artikels of toekomstige onderzoeken, zullen enkel gebeuren door middel van gecodeerde gegevens, die niet terug te brengen zijn naar u. De gegevens die de opdrachtgever krijgt, zullen hem dus niet in staat stellen jou of je patiënten te identificeren.

Wat zal er gebeuren met de informatie die tijdens de studie verzameld wordt?

Je deelname aan de studie betekent dat je persoonsgegevens en gegevens vanuit het EMD

- door de onderzoekers worden verzameld, en
- in gecodeerde vorm gebruikt worden door de opdrachtgever van het onderzoek.

De onderzoekers en de opdrachtgever mogen de gecodeerde persoonsgegevens alleen gebruiken voor onderzoeksdoeleinden in verband met wetenschappelijke publicaties in het kader van de studie waaraan je deelneemt. Indien ruimer gebruik van de gecodeerde gegevens gepland is, zal dat hieronder vermeld staan.

Bovendien kan de opdrachtgever externe onderzoekers (die niet betrokken zijn bij deze studie) toegang verlenen tot de gecodeerde gegevens. Indien een externe onderzoeker de gegevens wil gebruiken in onderzoek dat nog niet beschreven staat in dit document, moet dit onderzoek door een Ethisch Comité goedgekeurd worden.

Hoe zullen mijn gegevens verwerkt worden?

Uw gegevens zullen worden verwerkt overeenkomstig de Europese Algemene Verordening inzake Gegevensbescherming (AVG) en de Belgische Wetgeving betreffende de bescherming van natuurlijke personen met betrekking tot de verwerking van persoonsgegevens. KU Leuven is de verwerkingsverantwoordelijke voor uw gegevens.

De reden waarom wij persoonsgegevens en gegevens uit he EMD mogen verwerken, is dat we wetenschappelijk onderzoek verrichten en dat je toestemming hebt gegeven.

Heb ik toegang tot mijn gegevens die tijdens de studie verzameld en verwerkt zijn en kan ik ze rechtzetten?

Je hebt het recht om aan de onderzoekers te vragen welke gegevens over jou worden verzameld en waarvoor ze gebruikt worden in deze studie. Je hebt het recht om:

- Toegang te krijgen tot deze gegevens en ze na te kijken
- De verzamelde persoonsgegevens te ontvangen
- Correctie te vragen als ze niet juist zijn

Voor de gegevens verkregen vanuit het EMD is het niet mogelijk om:

- Correctie aan te vragen
- Gegevens te laten schrappen

Om te voorkomen dat de resultaten van de studie foutief geïnterpreteerd worden zijn deze beperkingen opgelegd. De gegevens van het EMD zijn namelijk, geanonimiseerde, patiëntgegevens en zijn essentieel in het beantwoorden van de onderzoeksvraag. Aanpassingen hieraan kunnen dit beïnvloeden.

Wie anders dan de onderzoekers en zijn personeel heeft toegang tot de gegevens?

Om de kwaliteit van de studie te controleren kan het gebeuren dat je gecodeerde gegevens of voor deze studie relevante informatie geïnspecteerd worden door andere mensen dan het studiepersoneel. Deze inzage gebeurt onder het toezicht van de onderzoekers en deze personen zijn gebonden aan het beroepsgeheim of via een vertrouwelijkheidsovereenkomst. Het kan gaan om:

- door de opdrachtgever aangeduid personeel (MONITORS en AUDITORS) en mensen of organisaties die diensten leveren aan of samenwerken met de opdrachtgever. Zij zullen echter nooit je naam en contactgegevens doorgeven aan de opdrachtgever.
- een onafhankelijke auditgroep
- personen aangeduid door het Ethisch Comité.

Indien nodig voor de studie mogen de gecodeerde studiegegevens naar andere landen binnen en buiten de Europese Unie (EU) worden gestuurd en worden nagekeken door:

- het/de Belgisch(e) evaluerend(e) Ethisch(e) Comité(s),
- externe onderzoekers,
- de opdrachtgever van de studie, door de opdrachtgever aangeduid personeel en mensen of organisaties die diensten leveren aan of samenwerken met de opdrachtgever, en/of
- bedrijven uit de groep van de opdrachtgever in België en in andere landen binnen en buiten de EU.

De Europese regelgeving en de Belgische wetgeving over gegevensbescherming leggen beperkingen op voor de overdracht van gegevens naar niet-EU landen. De opdrachtgever moet altijd verzekeren dat je gecodeerde studiegegevens gelijkwaardig beschermd worden bij overdracht naar een niet-EU-land. Indien de opdrachtgever hiervoor een overeenkomst over databescherming afsluit, kan een kopie van deze overeenkomst worden verkregen via de onderzoekers. Je kan altijd met de onderzoekers contact opnemen voor meer informatie over zulke overdracht.

Wat zal er gebeuren met de resultaten van de studie?

Na afsluiting van de studie zal een beschrijving en de resultaten van de studie gepubliceerd worden in gespecialiseerde medische tijdschriften. Een kopie van de wetenschappelijke publicatie of een voor een deelnemer begrijpelijke samenvatting, is verkrijgbaar via de onderzoekers of het studiepersoneel.

Zullen mijn gegevens gebruikt worden voor andere doeleinden dan de studie waaraan ik deelneem?

De resultaten van de studie zullen alleen worden gebruikt om een antwoord te geven op de wetenschappelijke vragen in deze studie.

Hoe lang worden mijn gegevens bijgehouden?

Na afloop van de studie zullen je gecodeerde gegevens minstens 20 jaar worden bijgehouden om de geldigheid van het onderzoek te verzekeren. Dat zal ook het geval zijn indien je voortijdig met je deelname aan de studie stopt.

> Indien u vragen hebt over hoe wij uw gegevens gebruiken of uw recht op inzage, correctie, eventueel stopzetting van de verdere verwerking wil uitoefenen, dan kan u hiervoor steeds terecht bij uw arts-onderzoeker op volgend contactadres: ann.li@kuleuven.be. Indien u naderhand nog bijzondere aandachtspunten heeft of klacht wenst neer te leggen, kan u terecht bij het privacy team van de KU Leuven op privacy@kuleuven.be

10. Wie heeft de documenten inzake de studie nagekeken en goedgekeurd?

De studiedocumenten werden nagekeken door een onafhankelijk Belgisch Ethisch Comité. De bevoegde ethische comités hebben als taak de personen die aan een studie deelnemen te beschermen. Je mag hun goedkeuring niet opvatten als een stimulans om deel te nemen aan de studie.

Om te onthouden

- ✓ Je deelname is vrijwillig; er kan op geen enkele manier sprake zijn van dwang. Voor deelname is jouw ondertekende toestemming nodig. Ook nadat je hebt getekend, kan je de onderzoekers laten weten dat je jouw deelname wilt stopzetten. De beslissing om al dan niet (verder) deel te nemen zal geen enkele negatieve invloed hebben op de relatie met de betrokken onderzoekers voor toekomstige samenwerkingen.
- ✓ De gegevens die in het kader van jouw deelname worden verzameld, zijn vertrouwelijk. Bij de publicatie van de resultaten is jouw anonimiteit, en die van de patiënten, verzekerd.
- ✓ Er zijn geen bijkomstige kosten bij het vervolledigen van de studie. Verplaatsingsonkosten zullen gecompenseerd worden.
- ✓ Indien je extra informatie wenst, kan je altijd contact opnemen met de onderzoekers of een medewerker van zijn of haar team.

Contact

Voor vragen met betrekking tot de rechten als deelnemer aan een studie, kunt u contact opnemen met de Ethische Commissie Onderzoek UZ/KU Leuven (e-mail: ec@uzleuven.be, Tel +32-16-348600

Voor vragen of opmerkingen met betrekking tot het onderzoek kan je zich wenden tot één van de volgende personen:

Bram Pussig
Tel.: +32 16 32 51 42
E-mail: bram.pussig@kuleuven.be

Prof. Catharina Matheï
E-mail: catharina.mathei@kuleuven.be

Dr. Mieke Vermandere
E-mail: mieke.vermandere@kuleuven.be

Prof. Bert Aertgeerts
E-mail: bert.aertgeerts@kuleuven.be

Geïnformeerde toestemmingsformulier voor deelname aan de studie: Implementatie studie om alcohol gerelateerde vroeg-detectie en kort advies op te schalen in de eerstelijnszorg met als doel overmatig alcohol te verlagen in de gemeenschap: Het PINO-project.

<u>Deelnemer/ vrijwilliger</u>

Ik verklaar dat ik geïnformeerd ben over de aard, het doel, de duur, de eventuele voordelen, de gevolgen van de studie en dat ik weet wat van mij wordt verwacht. Ik heb kennisgenomen van het informatiedocument.

Ik heb voldoende tijd gehad om het toestemmingsformulier te lezen, te bespreken met een vertrouwenspersoon en eventuele vragen te stellen.

Ik heb alle vragen kunnen stellen die bij me opkwamen en ik heb een duidelijk antwoord gekregen op mijn vragen.

Ik begrijp dat mijn deelname aan deze studie vrijwillig is en dat ik vrij ben mijn deelname aan deze studie stop te zetten zonder dat dit mijn relatie schaadt met het onderzoeksteam.

Ik begrijp dat er tijdens mijn deelname aan deze studie gegevens over mij zullen worden verzameld en dat de onderzoekers de vertrouwelijkheid van deze gegevens verzekeren overeenkomstig de Belgische wetgeving ter zake.

Ik begrijp dat ik bij deelname aan deze studie geen kosten heb.

Ik begrijp dat ik moet meewerken en de instructies van de onderzoekers en van het studiepersoneel rond de studie moet volgen en dat mijn deelname aan de studie zonder toestemming kan beëindigd worden om gelijk welke gerechtvaardigde reden.

Ik stem in met de verwerking van mijn persoonlijke gegevens volgens de modaliteiten die zijn beschreven in de rubriek over het verzekeren van de vertrouwelijkheid.

Ik ga akkoord dat de verzamelde antwoorden bewaard worden voor twintig jaar na het onderzoek waarna ze met de nodige maatregelen vernietigd zullen worden.

Ik heb een exemplaar ontvangen van de informatie aan de deelnemer en de geïnformeerde toestemming.

Naam, voornaam, datum en handtekening van de deelnemer:

<u>Onderzoeker</u>

Ik ondergetekende, onderzoeker, verklaar de benodigde informatie inzake deze studie mondeling alsook schriftelijk te hebben verstrekt evenals een exemplaar van het informatiedocument aan de deelnemer te hebben verstrekt. Ik heb nagegaan of de deelnemer de studie begrepen heeft.

Ik bevestig dat geen enkele druk op de deelnemer is uitgeoefend om hem/haar te doen toestemmen tot deelname aan de studie en ik ben bereid om op alle eventuele bijkomende vragen te antwoorden.

Ik bevestig dat ik werk in overeenstemming met de ethische beginselen en de Belgische wet van 25 mei 2018 inzake privacywetgeving.

Naam, voornaam, datum en handtekening van de onderzoeker:

Geïnformeerde toestemmingsformulier voor deelname aan de studie: Implementatie studie om alcohol gerelateerde vroeg-detectie en kort advies op te schalen in de eerstelijnszorg met als doel overmatig alcohol te verlagen in de gemeenschap: Het PINO-project.

Deelnemer/ vrijwilliger

Ik verklaar dat ik geïnformeerd ben over de aard, het doel, de duur, de eventuele voordelen, de gevolgen van de studie en dat ik weet wat van mij wordt verwacht. Ik heb kennisgenomen van het informatiedocument.

Ik heb voldoende tijd gehad om het toestemmingsformulier te lezen, te bespreken met een vertrouwenspersoon en eventuele vragen te stellen.

Ik heb alle vragen kunnen stellen die bij me opkwamen en ik heb een duidelijk antwoord gekregen op mijn vragen.

Ik begrijp dat mijn deelname aan deze studie vrijwillig is en dat ik vrij ben mijn deelname aan deze studie stop te zetten zonder dat dit mijn relatie schaadt met het onderzoeksteam.

Ik begrijp dat er tijdens mijn deelname aan deze studie gegevens over mij zullen worden verzameld en dat de onderzoekers de vertrouwelijkheid van deze gegevens verzekeren overeenkomstig de Belgische wetgeving ter zake.

Ik begrijp dat ik bij deelname aan deze studie geen kosten heb.

Ik begrijp dat ik moet meewerken en de instructies van de onderzoekers en van het studiepersoneel rond de studie moet volgen en dat mijn deelname aan de studie zonder toestemming kan beëindigd worden om gelijk welke gerechtvaardigde reden.

Ik stem in met de verwerking van mijn persoonlijke gegevens volgens de modaliteiten die zijn beschreven in de rubriek over het verzekeren van de vertrouwelijkheid.

Ik ga akkoord dat de verzamelde antwoorden bewaard worden voor twintig jaar na het onderzoek waarna ze met de nodige maatregelen vernietigd zullen worden.

Ik heb een exemplaar ontvangen van de informatie aan de deelnemer en de geïnformeerde toestemming.

Naam, voornaam, datum en handtekening van de deelnemer:

Onderzoeker

Ik ondergetekende, onderzoeker, verklaar de benodigde informatie inzake deze studie mondeling alsook schriftelijk te hebben verstrekt evenals een exemplaar van het informatiedocument aan de deelnemer te hebben verstrekt. Ik heb nagegaan of de deelnemer de studie begrepen heeft.

Ik bevestig dat geen enkele druk op de deelnemer is uitgeoefend om hem/haar te doen toestemmen tot deelname aan de studie en ik ben bereid om op alle eventuele bijkomende vragen te antwoorden.

Ik bevestig dat ik werk in overeenstemming met de ethische beginselen en de Belgische wet van 25 mei 2018 inzake privacywetgeving.

Naam, voornaam, datum en handtekening van de onderzoeker:
